# Importance of trust and personal responsibility issues in sculpting the health knowledge landscapes

**DOI:** 10.3325/cmj.2019.60.46

**Published:** 2019-02

**Authors:** Predrag Pale

**Affiliations:** University of Zagreb, Faculty of Electrical Engineering and Computing, Zagreb, Croatia *Predrag.Pale@FER.hr*

The internet and personal computers have radically changed the ways and dynamics of acquiring information and knowledge. Information with greater importance to the user has a stronger impact, and health information is particularly valued by users. The internet is becoming the primary source of health related information for general public (1), especially in younger generations (2). However, it is loaded with inaccurate, outdated, wrong, and false information. SPAM, hoax, fake news (3), and hate wars are just some of the phenomena plaguing knowledge landscapes that could bring so much good to individuals and humanity as a whole. This article analyzes knowledge flow and exchange, motivations, and behavior of users and producers, as well as problems with information validity. Finally, it offers a view on the importance of trust and personal responsibility issues.

## THE KNOWLEDGE LANDSCAPE CREATION AND USE

**Figure 1:**
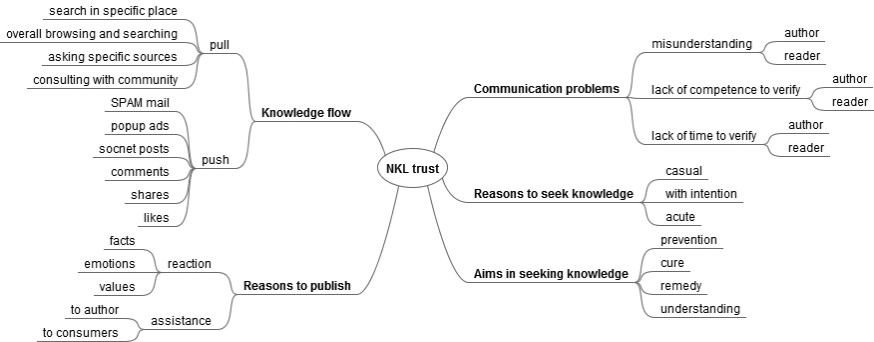
Understanding the knowledge landscape creation and use.

Before the importance of trust and issues of personal responsibility are addressed, it is important to understand why people seek information and how and when they do it. How does information get to the users? How and why do authors, professionals and laymen, create and disseminate information (Figure 1)?

### The knowledge flow and exchange methods

There are two major ways information gets to the user: pull and push mode. In the pull mode, the user is actively seeking information. This can be accomplished in several ways. Browsing the overall knowledge space is the common method of choice when the user does not have a specific question but is rather trying to identify potential topics and sources in a broader area of his interest. Search is focused on specific sources of information when the user has a very specific question. A novel way is consulting with a community by posting questions to specific user groups.

The push mode is when information gets to the user without his explicit request or inquiry. Traditionally it is via e-mails, sometimes targeted and sometimes in the form of SPAM or Hoax messages (4). More recent forms are ads and pop-up ads on web sites and various services including social networks. However, the most common medium today are interactions in social networks. There are several ways to exchange information in social networks. The most direct way is by posting either own thoughts or compiling other people’s thoughts. A less direct way is by commenting someone else’s post. An even less direct way is by sharing someone else’s post. While sharing, just like when forwarding an email, the sharer also communicates a co-message saying: “This information is important.” If no comment is attached, it might not be obvious whether the sharer agrees with the information or not. Finally, the most indirect way of sharing information is by “liking” someone’s post, which is a highly ambiguous act since the emoticons like “like,” “smile,” “sad,” or “angry” are too uninformative to adequately convey the “liker’s” message.

### The reasons to seek or accept health knowledge

Regardless of whether they use push or pull model, users seek or accept health related information for one of the three reasons: casual motivation, some intention, or acute need. Casual motivation means the user has a mild, non-urgent, and weakly focused interest in a health related topic, range of topics, or knowledge area. For example, fitness, healthy nutrition, maintaining memory are frequently interesting types of information but are rarely actually used or strictly followed. They rarely and mildly modify user’s decisions and behavior. The sources are general, broad, and not very specific. Some intention is shown when user wants to find a solution for a specific problem like weight-loss, kidney stones, or improving eyesight. In this situation, the user actively browses or searches for required information from a narrowed list of sources and posts questions in dedicated communities. Acute situations mean there is some urgency to solve a health problem. These are usually related to pains, grave diseases like cancer, and injuries that are preventing the user from a timely obligation or duty. In acute situations, both strategies are applied: broad survey and consultation as well as targeted searching and discussion. For the latter, the reliability of information source and personal trust are very important.

### Aims in seeking knowledge

The aims of seeking health information are also multifold. Seeking a cure is usually focused on seeking a solution: recipe, pill, or therapist. It is usually not focused on seeking to understand the problem and its solution. Another aim is seeking a remedy. Remedies usually do not cure, but rather relieve the symptoms. Again, no deeper or broader involvement with the topic is intended, nor is broader information expected. It is only when the aim is to understand both the problem and solution that more detailed and broader information is expected and more time and effort to process them are invested. The fourth aim is to prevent the problem. It can be done in the same fashion as seeking the cure or like seeking understanding. The required reliability of the information source depends on the intention, where seeking cure and understanding have the most demanding requirements and prevention has the least demanding ones.

### Creating information

In the pre-internet age, information was predominantly produced by a few renowned authorities. While this did not guarantee absolute truth or information quality, it did significantly restrict low-quality or false information. This concept of publishing has radically changed. Currently, anyone can send any information to anyone. It is the intended recipient who actually needs to make an effort not to receive information, which is quite a novel concept, inverting the logic of previous times.

Since no one has to approve or filter what will be published and disseminated, the information is not verified or carefully worded and framed. Simultaneously, the dominant culture values speed above accuracy and visual appeal above substance (5). These forces combined result in the low quality of information produced. The content can be original or created by processing thoughts of other authors: re-telling, explaining, and compiling.

### Sculpting the knowledge landscape

Besides generic information created by the author as original producer, the landscapes are formed by sharing, commenting, and liking. In order to understand these processes it is important to understand the reasons why an author is publishing. There are two main groups of reasons, to react and to assist.

To react to the facts published can mean to oppose them, offer different facts, generalize them, or provide more details or explanations. There can also be a reaction to emotions presented in original publications, which can be intensified, denied, or (re)directed. Finally, the most intensive reactions are to the values presented in the original publications. Due to limited expressibility of text, values are misunderstood or misinterpreted, which can lead to inappropriate reactions, distorting the knowledge landscape. The second group of reasons is to assist either the audience or the original author. In both cases, the intention is to provide information, clarify, correct, educate, or encourage.

### The knowledge space plague

Some published information is outright lies. Reasons why people intentionally publish lies vary from malicious intent, joking, experimenting, gaining popularity, to fear from truth. Lies, semi-truths, spins, gossips, and other forms of faulty information are not new and are not medium dependent. However, the internet is now experiencing, or generating, a flood of new phenomena: identity theft, impersonation, fake news, fake pictures and fake videos (6). From one point of view, this is very bad news since nothing can be taken at its face value. There is no type of information, form, source, or media that automatically guarantees the validity and truthfulness of information. Everything needs to be verified. On the other hand, this might be good news if users’ adopt an attitude, a doctrine, that nothing is true unless proven. Users could reduce their stress and strain by considering most information as fun or trash but putting an effort into analyzing only really important information. This could also mean that small, but essential, circles of trust could be formed.

### Problems with the quality of information

However, even if a piece of information is not a lie, it can be foggy, wrong, or outdated. It can also be inappropriate for a specific audience. The metadata indicating information’s intended audience and context could sometimes be decoded from the location where the information was published, but when information is shared, compiled, or processed these metadata are lost.

A significant number of communication problems stems from a misunderstanding between the author or publisher and the end user. The reasons for misunderstanding are authors’ lack of competence to convey the information clearly, the fact that information was not intended for a particular audience, or information distortion on the way from the original author to the end user.

Before a piece of information is forwarded, shared, or liked, its validity should be verified. The same is true for receiving information. However, this requires knowledge, motivation, and time. When attractive information gets to the attention of a person, he or she cannot resist immediately sharing or liking it. There is usually lack of time, or perception of lack of time, to verify the information. The more the information is in line with the user’s needs, values, beliefs, or attitudes, the weaker is his or her motivation to thoroughly verify the information.

## THE TRUST

The more acute and personally important health-related information is to the user, the more will he or she rely on information coming from a trusted source (7). The key to understanding information validity and trust is the users’ insufficient competence to judge the information validity. They simply have to trust the source. Traditionally, users are inclined to trust authorities. These are in the first place renowned institutions, preferably public and independent. Private institutions with long tradition of trustworthiness are also important. But people like to trust people: authorities by education, affiliation, or track record. And they do like to trust people they personally know.

## PERSONAL RESPONSIBILITY

Users are responsible for analyzing the information, its source, and its path from the source to users. However, the key responsibility lies with those creating and publishing the information. Even if the information is intended for a broad public and is written in simple language, authors should apply basic principles of professional and scientific publishing: clearly cite the sources, provide reference for every information, and avoid non-specific terms like “many,” “always,” “never,” “probably,” etc. This might be a difficult request, especially for popular texts and social networks’ culture, but it is becoming necessary because of the trust users place in original authors.

While responsibility might be obvious in cases of original authors, issues arise when other peoples’ information is being conveyed, interpreted, compiled, or commented. It is dangerous to presume that the sole responsibility for the information validity is with the original author. Bearing in mind that end users are less competent to validate the original information, the “intermediate authors” have to be aware that they will most likely be viewed as the one with more competence than the user. The first step is to validate the original information. If the intermediate authors are not fully sure if the original information is valid, they should clearly state so. The second step is to state the personal competence to validate the original information.

The same is true for other forms of intermediate authorship or publishing: sharing, commenting, and liking, when intermediate authors are even more likely to assume that they are not responsible for the validity of original information. However, this is wrong because end users will first check who the last information sender was. Intermediate authors should validate the information before they forward it, regardless of the medium or method: forwarding mail, posting opinion, sharing post, commenting or liking it. The most difficult task is to convince people of their responsibility for mere liking of a message. Their awareness could be raised by demonstrating how a simple “like” can mean a host of different things to different users: “I agree,” “It is important,” “it is interesting,” “I support the author (not the topic),” etc. Also, when an intermediate author “likes” someone’s reaction to an unfortunate event with an “angry face,” does that mean he or she is opposing the unfortunate event or the reaction of the original poster?

### Ways toward secure and trusted sources

The solution is, of course, in raising awareness and education: information and media literacy. The key approach would be focusing on information users and authors, predominantly health professionals, because they are most frequently viewed as a competent and trusted source of information. The accompanying challenge is to motivate and convince authors, especially intermediate authors to be careful and conscientious every time before they hit “send,” “post,” or “like” button.

The users’ education would be most successful if they could be engaged in detecting the validity of interesting, intriguing, or controversial information relevant to them personally. The dynamics of modern life is calling for urgency, being first before being right. Authors are invited to investigate all possible negative effects of seemingly innocent posts without measures of precaution. Also it could be beneficial to exercise seeking faulty information coming from sources authors trust.

### CONCLUSION

As the proportion of false information in knowledge landscapes continues to grow, the issue of information validity becomes paramount. Users turn to trusted sources of information, preferring people they know or perceive to have authority. For this reason the responsibility of authors to be very clear and precise in their original content calls for adopting principles applied in professional and scientific publications. However, the biggest challenge is to convince intermediate authors, those who convey somebody else’s original information, that they too have to carefully validate original information and be very clear about the co-message they send by conveying.

It is a question whether it is feasible to raise users and authors’ awareness to the level where major accidents can be prevented. The dynamics and directions in which global information space is developing are unpredictable. However, health and education professionals have the responsibility to try and do their best. This means information and media literacy needs to be a crucial component of general education from the earliest age (K1-4). It also has to be part of professional education from day one and it has to be checked all the time.
